# Plasma Exosomal Non-Coding RNA Profile Associated with Renal Damage Reveals Potential Therapeutic Targets in Lupus Nephritis

**DOI:** 10.3390/ijms24087088

**Published:** 2023-04-11

**Authors:** Ana Flores-Chova, Olga Martinez-Arroyo, Angela L. Riffo-Campos, Ana Ortega, Maria J. Forner, Raquel Cortes

**Affiliations:** 1 Cardiometabolic and Renal Risk Research Group, INCLIVA Biomedical Research Institute, 46010 Valencia, Spain; afloreschova@gmail.com (A.F.-C.); omartinez@incliva.es (O.M.-A.); 2 Millennium Nucleus on Sociomedicine (SocioMed) and Universidad de La Frontera, Doctorado en Ciencias Medicas, Temuco 4780000, Chile; angela.riffo@ufrontera.cl; 3 Department of Computer Science, ETSE, University of Valencia, 46010 Valencia, Spain; 4 CIBERCV (CIBER of Cardiovascular Diseases), 28029 Madrid, Spain; 5 Internal Medicine Unit, Hospital Clinico Universitario, 46010 Valencia, Spain; 6 Department of Medicine, Faculty of Medicine, University of Valencia, 46010 Valencia, Spain

**Keywords:** systemic lupus erythematosus, exosomes, non-coding RNA, RNA sequencing, bioinformatics enrichment analysis

## Abstract

Despite considerable progress in our understanding of systemic lupus erythematosus (SLE) pathophysiology, patient diagnosis is often deficient and late, and this has an impact on disease progression. The aim of this study was to analyze non-coding RNA (ncRNA) packaged into exosomes by next-generation sequencing to assess the molecular profile associated with renal damage, one of the most serious complications of SLE, to identify new potential targets to improve disease diagnosis and management using Gene Ontology (GO) and the Kyoto Encyclopedia of Genes and Genomes (KEGG) analysis. The plasma exosomes had a specific ncRNA profile associated with lupus nephritis (LN). The three ncRNA types with the highest number of differentially expressed transcripts were microRNAs (miRNAs), long non-coding RNAs (lncRNAs) and piwi-interacting RNAs (piRNAs). We identified an exosomal 29-ncRNA molecular signature, of which 15 were associated only with LN presence; piRNAs were the most representative, followed by lncRNAs and miRNAs. The transcriptional regulatory network showed a significant role for four lncRNAs (LINC01015, LINC01986, AC087257.1 and AC022596.1) and two miRNAs (miR-16-5p and miR-101-3p) in network organization, targeting critical pathways implicated in inflammation, fibrosis, epithelial–mesenchymal transition and actin cytoskeleton. From these, a handful of potential targets, such as transforming growth factor-β (TGF-β) superfamily binding proteins (activin-A, TGFB receptors, etc.), WNT/β-catenin and fibroblast growth factors (FGFs) have been identified for use as therapeutic targets of renal damage in SLE.

## 1. Introduction

The analysis of non-coding RNA (ncRNA) profiles as biomarkers of disease state and progression and for monitoring therapy response has attracted increasing research interest over the past decade. These ncRNAs can be divided into different subtypes according to their size: small ncRNAs (sncRNAs) < 200 nt that include transfer RNA (tRNA), Y-RNAs, small nuclear ribonucleic acid RNA (snRNA), nucleolar RNA (snoRNA), piwi-interacting RNA (piRNAs) and microRNAs (miRNAs), and ncRNAs longer than 200 nt, such as long non-coding RNA (lncRNA) and ribosomal RNA (rRNA) [1]. ncRNAs are recognized as essential functional molecules tissue and cell-specific levels, regulating key genes involved in homeostasis, development and disease [1]. Numerous studies of ncRNAs highlight the close connection between ncRNA dysregulation and the pathogenesis of many human disorders, including systemic lupus erythematosus (SLE) [2,3,4,5]. While lncRNAs and miRNAs have garnered the most attention among the ncRNAs, recent research has shown increasingly diverse functions for other ncRNA biotypes.

SLE is a chronic autoimmune disease in which the immune system mistakenly attacks healthy tissues, causing extensive inflammation and tissue damage in the affected organs. One of the most severe organ manifestations of SLE is lupus nephritis (LN). Due to the lack of knowledge of the exact mechanisms that lead to the appearance of lupus flares affecting condition patient prognosis, together with progress in the field of high-throughput sequencing technology, a wide variety of ncRNAs have been found associated with SLE. Well-studied lncRNAs and miRNAs are associated with disease activity, to discriminate LN and response to therapy [6,7,8]. Y-RNAs are gaining attention in autoimmunity, as potential biomarkers and their role in immune cell communication [9,10]. In a recent study, Yang P et al. demonstrated that tRNA-derived small noncoding-RNA signatures can be employed as noninvasive biomarkers for the efficient diagnosis and prediction of nephritis in SLE [11].

Circulating ncRNAs can be detected in biofluids bound to RNA-binding proteins or packaged into extracellular vesicles (EVs), such as exosomes, functioning as paracrine effectors in the crosstalk between cell types [12]. Previous studies have established exosomal ncRNAs as biomarkers for diagnosis, prognosis and for monitoring therapy in immune diseases [9,13,14,15], and exosomal miRNA are the most studied in the SLE setting. Our group has identified exosomal miR-146a-3p levels associated with albuminuria, activity changes and disease flares in LN [4]. Garcia-Vives et al. showed that urinary exosomal miR-135b-5p, miR-107 and miR-31 regulate LN renal recovery by HIF1A inhibition [16]. Tan et al. demonstrated that downregulated exosomal miR-451a expression is correlated with SLE activity and renal damage, and is involved in cell communication between mother cells and T or B cells [17]. However, analyses of the specific function of the global exosomal ncRNA profile in SLE and the mechanism of involvement in related pathological changes such as LN remain underexplored.

The aim of this study is to identify an exosomal expression profile of ncRNAs and their biological pathways regulated in SLE using full high-throughput sequencing and bioinformatics analysis. We constructed exosomal lncRNA–mRNA and miRNA–mRNA interaction networks to understand the mechanisms of renal damage in SLE at the molecular level and to develop a new potential therapeutic approach.

## 2. Results

### 2.1. Study Population

The study cohort included 96 samples from SLE patients, 23 of which had an LN diagnosis, and 25 healthy controls. General patient characteristics are shown in Table 1. Pathological groups and controls were matched for age and sex percentage. Patients with LN had augmented levels of the dsDNA and SLEDAI index (*p* < 0.01). In addition, altered renal function in LN was observed, as indicated by low eGFR values and high proteinuria levels (*p* < 0.01).

### 2.2. Proportions of Differentially Expressed RNA Types in Systemic Lupus Eryhtematosus with or without Lupus Nephritis According to Biofluid

**Figure 1:**
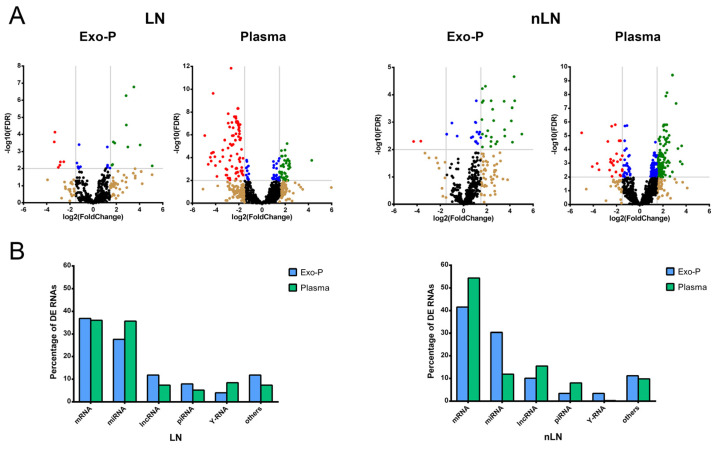
Differentially expressed RNAs in SLE patients with or without renal damage in each biological fraction compared to control subjects. (**A**) Volcano plot depicts significantly altered RNAs found in LN or nLN. Each dot represents an RNA; non-significant false discovery rate (FDR > 0.05) and log2 fold-change ≥−1.5 or ≤1.5) in black, log2 fold-change ≤−1.5 or ≥1.5 in brown, significant FDR in blue and significant FDR and log2 fold-change ≥1.5, green (up-regulated) or ≤−1.5 in red (downregulated). The threshold dotted line for −log10(FDR) it was <0.05 and for log2 fold-change was ≤1.5 or ≥−1.5, and; (**B**) Bar graph of RNA subtype percentages according to the two biofluids in LN and nLN. DE: differentially expressed; Exo-P: plasma exosomes; LN: lupus nephritis; nLN: non lupus nephritis. lncRNA: long non-coding RNA; miRNA: microRNA; mRNA: messenger RNA; piRNA: PIWI-interacting RNA.

As shown in the volcano plot, the analysis of RNA subtypes in SLE patients with LN or without LN (nLN) for each biofluid identified more significant RNAs differentially expressed (DE) (FDR < 0.05) in plasma than in the exosome fraction in both groups compared to control subjects (Figure 1A). In plasma samples, significant RNA showed higher fold change (FC) than in those in exosomes and had the most of DE ncRNA downregulated in LN and up-regulated in nLN. In LN, however, the situation was reversed: Most ncRNAs were up-regulated in both groups. Analyzing the number of DE transcripts (considering *p*-value < 0.05) according to biofluid, in Exo-P, the protein-coding genes corresponded to approximately 42% of the total transcripts, followed by 26% miRNA and 11% lncRNA. For plasma samples, we observed 57% for protein-coding genes, 14% lncRNA and 11% miRNAs (Figure 1B). Interestingly, irrespective of the presence of renal damage, the percentages of all RNA subtypes showed an inverse pattern. Taking as an example the three most representative RNA subtypes, mRNAs had similar percentages between biofluids in LN but were higher in plasma in nLN; miRNAs were higher in plasma in LN than Exo-P, but lower in nLN; and Exo-P lncRNA was augmented in LN, but was lower in plasma from nLN patients. Furthermore, Y-RNAs were more representative in the LN than the nLN group.

### 2.3. Plasma Exosomal ncRNA Signature Sssociated with Renal Damage

**Figure 2:**
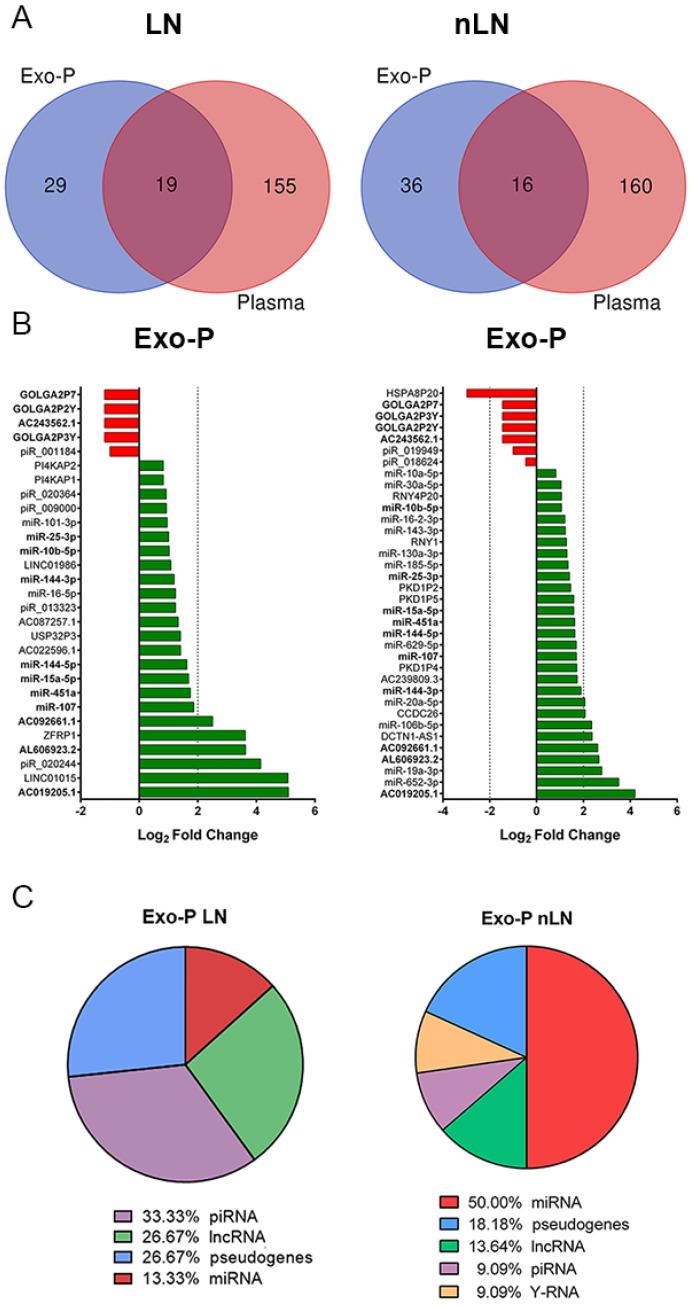
Differentially expressed ncRNAs profiles in plasma exosome fraction of SLE patients with or without LN compared to control subjects. (**A**) Venn diagram shows the overlap among biological fractions. (**B**) Diverging bar charts show the fold-change expression of the exosomal non-coding RNAs signature in both groups: upregulated are in green and downregulated in red. Common NcRNAs in both signatures are in bold. logFC: logarithm 2 base fold-change; (**C**) Proportions of DE ncRNA biotypes in plasma exosomes from SLE patients with LN or without (nLN). Exo-P: plasma exosomes; LN, lupus nephritis; lncRNA: long non-coding RNA; miRNA: microRNA; piRNA: PIWI-interacting RNA; nLN: non lupus nephritis.

The Venn diagram obtained from among the biological fractions showed very limited overlapping between plasma and exosomal fractions, revealing 19 of these 203 DE ncRNAs in LN and 16 of the 212 in nLN (Figure 2A). Focusing on the exo-P fraction, diverging charts showed that most of the DE ncRNAs were up-regulated in Exo-P in both SLE groups: 9 of the 29 ncRNAs were miRNAs, 8 were pseudogenes, 7 were lncRNAs (24%), and 5 were piRNAs in LN (Supplementary Table S1). For exosomal ncRNA from nLN, 18 of the 36 were miRNAs, 8 were pseudogenes, 6 were lncRNAs, 2 were piRNAs and 2 were Y-RNAs (Figure 2B). From these two exosomal ncRNA profiles, ncRNAs common to both signatures (highlighted in bold) were excluded from the subsequent enrichment analysis, so piRNAs (33%), lncRNAs (27%) and miRNAS (13%) were the most abundant in LN (Figure 2C). From these 15 exosomal ncRNAs from LN, we included 2 up-regulated miRNAs (miR-101-3p and miR-16-5p) and 4 up-regulated lncRNAs (LINC01986, AC087257, AC022596.1 and LINC01015) to identify their potential predicted targets. Before, we verified that there were no statistical differences in the expression levels of these six ncRNAs in patients with LN, but that they were associated with kidney damage (Supplementary Figure S1). As there are no specific databases to predict the targets of piRNAs, these were not included in the enrichment analysis.

### 2.4. Differentially Expressed miRNA-mRNA Network from Patients with Nephritis

**Figure 3:**
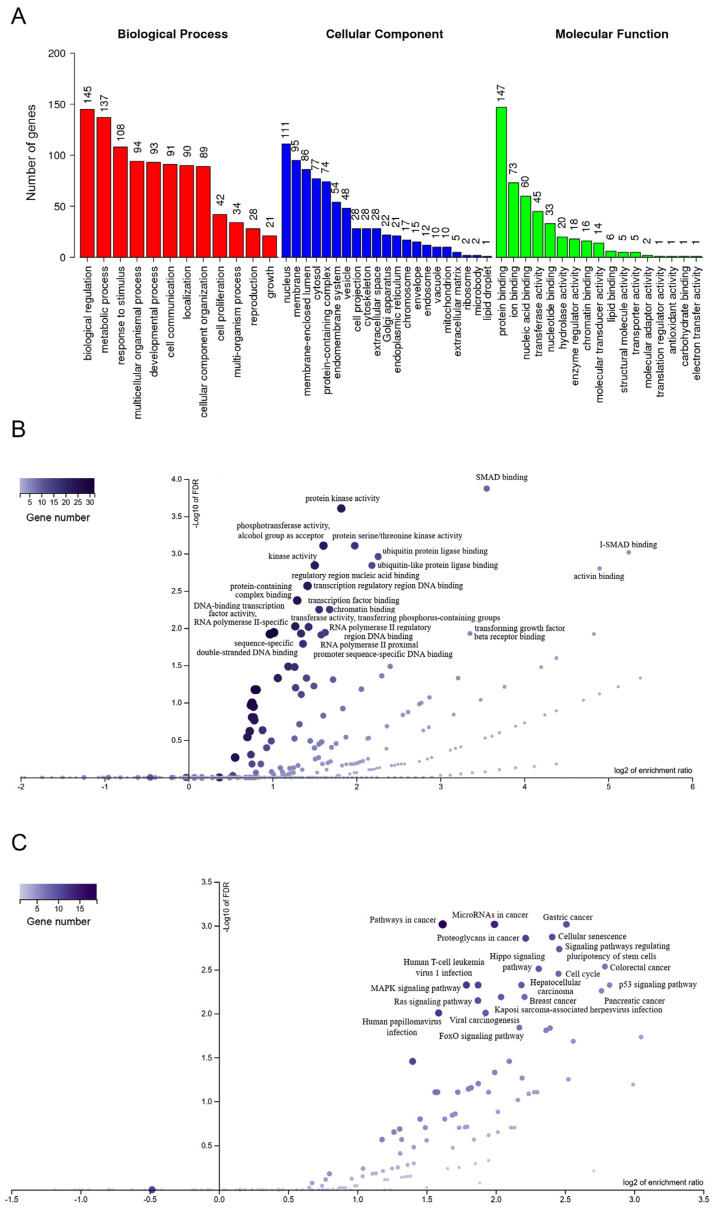
Functional enrichment analysis of the DE exosomal miRNA targets in SLE patients with LN. (**A**) GO analysis of the DE targeted genes. The vertical axis indicates the number of targeted genes in a particular hierarchical cluster in biological process, cellular component and molecular function. (**B**) Volcano plot of GO terms enrichment analysis, according to the enrichment ratio (horizontal axis) and –log10(FDR) (vertical axis). The top 20 significant GO terms are described. (**C**) Volcano plot of the KEGG pathway enrichment analysis. The top 20 significant KEGG pathways are described. The size and color of the dot (enriched gene set) is proportional to the size of the category.

The two DE miRNAs and four lncRNA established in the molecular signature associated with LN were selected and the potential predicted target mRNAs were identified, creating the two networks (Supplementary Table S2). First, hierarchical clustering of miRNA target genes was conducted according to the biological process (BP), cellular component (CC) and molecular function (MF) categories of GO terms. Twelve BP, 21 CC and seventeen MF GO terms were found. BP terms included metabolic process, cell communication and cell proliferation. Vesicle, cell projection, cytoskeleton, golgi apparatus and endoplasmic reticulum and other GO terms were included among the CC. MF contained protein, ion, chromatin and lipid binding, and antioxidant activity (Figure 3A). Next, a volcano plot for the top 20 GO terms was performed (Figure 3B), revealing SMAD binding, activin binding Type I transforming growth beta receptor (TGFBR) binding and activity, beta catenin (β-catenin) binding and ubiquitin binding as having the highest enrichment ratios. A volcano plot for the top 20 KEGG pathways was also performed to reveal pathways involving the DE genes targeted by miRNAS in LN (Figure 3C). A KEGG pathways analysis showed that some pathways participated in the development of LN, such as inflammation (p53, MAPK and Ras signaling pathways), cell senescence and signaling pathways regulating stem-cell pluripotency.

**Figure 4:**
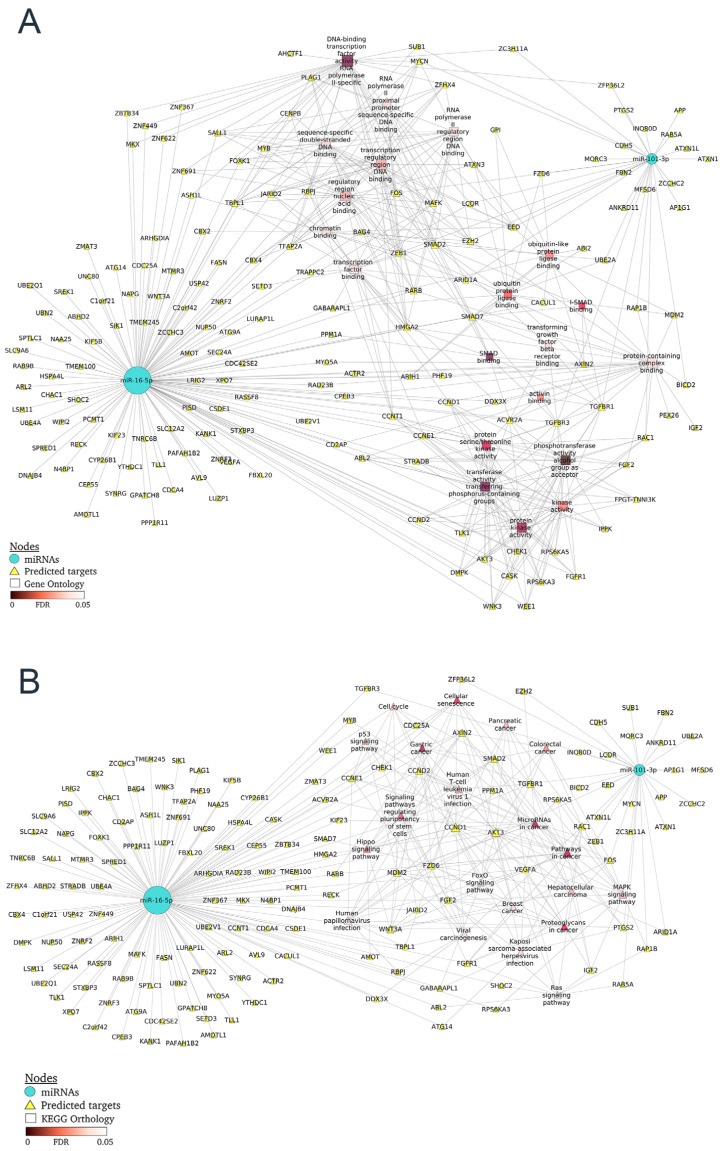
Regulatory networks of miRNAs–mRNAs for exosomal fraction in LN. (**A**) The two miRNAs (miR-16-5p and miR-101-3p) blue circles and their predicted targeted genes (yellow triangles) are shown together with the top 20 GO terms whose color is proportional to the FDR value, and (**B**) the top 20 KEGG pathways.

As a next step, the miRNA–mRNA networks together with GO terms or KEGG pathways were constructed for LN (Figure 4A and B, respectively). GO terms associated with TGFBR, SMAD and activin binding share the most gene targets. For the catenin binding, AXIN2, CD2AP, CDH5, KANK1, SALL1 and SMAD7 are annotated genes of our network included in this pathway. Finally, the miRNA–mRNA network together with KEGG pathways highlighted p53 signaling, which included cyclins and checkpoint kinase among other targets; signaling pathways regulating stem-cell pluripotency (FGF2, FGFR1, WNT3A, ACVR2A and SMAD) and cell senescence (cyclins, TGFBR1, SMAD7, AKT3, among others). Finally, the LN protein–protein interaction network allowed us to identify hub nodes according to confidence score: WNT3A, fibroblast growth factors (FGF2 and FGFR1), cyclins (CCND1, CCND2 and CCNE1), ACVR2A, SMAD7 and TFGB receptors (Supplementary Figure S2), which could be considered potential therapeutic targets related to the development of LN.

### 2.5. Regulation Networks of lncRNA–mRNA from Patients with Nephritis

**Figure 5:**
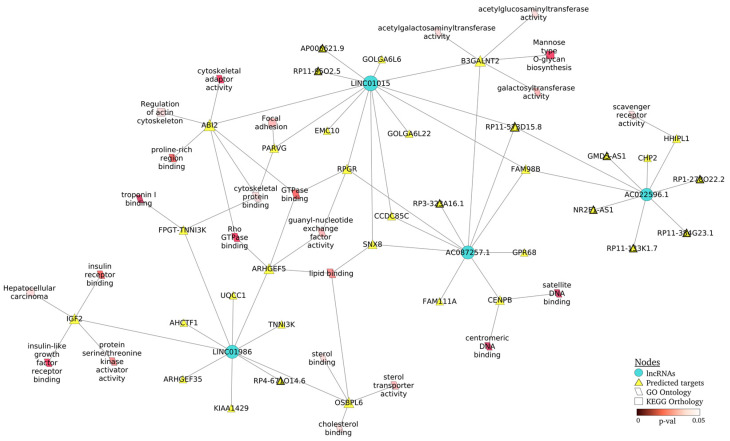
Regulatory network of lncRNAs–mRNAs for exosomal fraction in LN. The four lncRNAs (LINC01986, AC087257.1, AC022596.1 and LINC01015) represented as blue circles and their predicted targeted genes (yellow triangles) are shown, together with the top GO terms (parallelogram) and KEGG pathways (rectangle) whose color is proportional to the *p* value. Yellow triangles with bold border are lncRNAs as targets.

Next, we examined the details of the lncRNA–mRNA interaction network in LN (Figure 5), including the top GO terms (parallelogram form) and KEGG pathways (triangle form). We observed that LINC01015 and LINC01986 regulated most of the targets included in the biological pathways, highlighting Rho GTPase binding and GTPase binding (ABI2, ARHGEF5 and RPGR), cytoskeletal function (ABI2, FPGT-TNNI3K and PARVG) and insulin-like growth factor receptor binding (IGF2) as GO terms, as well as focal adhesion (PARVG) and regulation of actin cytoskeleton (ABI2) as KEGG pathways. In addition, we could observe that lncRNA not only regulated coding targeted genes, but also others lncRNAs (triangles with bold border, Figure 5).

## 3. Discussion

The incomplete understanding of the exact mechanisms that lead to the appearance of lupus flares that affect patient prognosis, together with the advances in the field of high-throughput sequencing technology, have resulted in the discovery of a wide variety of SLE-associated ncRNAs. The present study identified a specific exosomal 15-ncRNA profile associated with renal damage in SLE, where piRNAs, lncRNAs and miRNAs were the most representative small RNA biotypes. The transcriptional regulatory network identified four lncRNAs (LINC01015, LINC01986, AC087257.1 and AC022596.1) and two miRNAs (miR-16-5p and miR-101-3p) as playing a significant role in interaction network organization. In addition, enrichment over-representation analysis, with GO terms and KEGG pathways, detected critical pathways implicated in inflammation, fibrosis, epithelial–mesenchymal transition and an actin cytoskeleton that identifies a handful of potential targets, such as the TGF-β superfamily (activin-A, TGFB receptors…), WNT/β-catenin and FGFs, which may be useful as therapeutic targets of renal damage in SLE.

Analyzing DE RNA in SLE patients with or without LN according to biofluid origin, we observed that the percentage of DE RNAs in each biotype changed in plasma compared to Exo-P fraction in both pathological groups. For the three most representative ncRNA biotypes (miRNA, lncRNA and piRNA) plasma fraction had a higher percentage of DE miRNAs than Exo-P, while lncRNA and piRNAs were augmented in Exo-P of LN patients. However, these ncRNA percentages were inverse in SLE patients without LN. This fact, together with the low overlapping of DE RNAs between the two biofluids in the SLE groups, supports the idea of selective sorting of specific ncRNAs into exosomes under pathological condition, as previous results have shown [18,19,20].

The molecular exosomal ncRNA profiles in SLE patients associated or not with LN identified 29 ncRNA and 36 ncRNA signatures, respectively, in which the most DE ncRNAs were up-regulated in exosomal fraction. In the specific 15-ncRNA profile associated with LN, the most representative biotypes were piRNAs, lncRNAs and miRNAs. The two miRNAs identified in our molecular profile were miR-101-3p and miR-16-5p. A previous study has shown that up-regulation of miR-101 inhibits acute kidney injury and chronic kidney disease transition by regulating epithelial–mesenchymal transition [21]. Another work demonstrated that miR-101-3p is a regulator of renal interstitial fibrosis inhibiting TGF-β1-induced tubular EMT by targeting TβR-I [22]. Zhao et al. revealed that miR-101-3p negatively regulates inflammation in SLE via MAPK1 targeting and blocking the NF-κB pathway [23]. For miR-16-5p, previous results showed that macrophage-enriched miR-16-5p is a potential urinary biomarker of acute kidney injury in renal transplant recipients [24], and NEAT1/XIST/KCNQ1T1-miR-27a-3p/miR-16-5p-ATF3 might be a potential RNA regulatory pathway to regulate the disease progression of early diabetic nephropathy [25].

Enrichment analysis using GO annotation and KEGG pathways showed biological functions enriched in response to renal damage in plasma exosome fraction, allowing us to identify critical pathways regulated by the ncRNA profile. The miRNA–mRNA interaction network constructed for LN targeted critical pathways involved in inflammation (p53, MAPK and Ras signaling), fibrosis (TGF-β superfamily), epithelial–mesenchymal transition (WNT/β-catenin, FGFs) and cell senescence identified potential targets for LN progression and treatment such as activin-A, TGF-β receptors, WNT/β-catenin and FGFs.

Previous studies have demonstrated that the TGF-β superfamily plays an essential role in the regulation of immune responses contributing to or protecting against immune diseases [26]. As an example, activin-A is implicated in the pathology of human autoimmune diseases [27]. In the case of human rheumatic diseases, increased serum levels of activin-A were observed in SLE [28,29], and Kadiombo et al. showed augmented urinary levels of activin-A, but not protein or serum levels, in MRL-lpr mice, and suggested that infiltrating macrophage-derived activin-A could be involved in the progression of renal injury in this model of LN [30]. Tubulointerstitial lesions play a significant role in the LN progression, resulting in decreased renal function [31,32]. Recently growing evidence has demonstrated that the epithelial–mesenchymal transition (EMT) of renal tubular epithelial cells has been associated with susceptibility to LN [33,34]. Fu et al. found that the chemokine fractalkine plays an essential role in EMT progression and development of tubulointerstitial lesions in a murine model of LN, most likely through activation of the Wnt/β-catenin pathway [35]. A recent work by Wang et al. underscores the key role of miRNA-671-5p in mediating Wnt/β-catenin-triggered podocyte damage [36]. These data support the importance of the biological pathways identified in our miRNA–mRNA network in the development of renal damage and pinpoint the genes associated as potential targets for LN progression.

Moreover, the lncRNA–mRNA interaction network showed additional biological pathways associated with LN progress, such as focal adhesion and actin cytoskeleton, mediated by Rho GTPase binding and GTPase binding, identifying ABI2, PARVG and ARHGEF5 as potential targets. It is well established that the correct function of glomerular filtration relies on podocyte adhesion both at the interface with the extracellular membrane and at cell junctions [37]. The Rho family of small GTPases (Rho GTPases) closely regulates actin cytoskeleton and performs diverse cellular functions such as adhesion, migration and spreading in podocytes [38]. ARHGEF5, a member of the Rho guanine nucleotide exchange factor (GEF), is induced during the TGF-β-induced mesenchymal transition of endothelial cells [39]. Members of the Parvin family, including PARVG, are actin-binding proteins associated with integrin-linked kinase signaling and focal contacts. A recent work by Rogg et al. demonstrated their crucial role in the adaptive mechanisms of podocyte integrin adhesion complexes to prevent podocyte detachment in glomerular disease [40]. ABI2 is a component that forms a stable complex with WAVE proteins, which are involved in the connection of the membrane with actin cytoskeleton in podocytes [41].

The ncRNA signature also includes four piRNAs. In recent years, piRNAs have gained prominence as modulators of disease pathogenesis [42], and a recent systematic review analyzed the role of EV-derived piRNA in disease progression [13]. Only piR-20244 was associated with the GO terms mitochondrial inner membrane and cytochrome-c oxidase activity through the piRNAdb database. However, the current lack of guidelines on piRNA bioinformatics analysis has hindered progression in the discovery of piRNAs, their targets and the biological pathways that they regulate.

A key feature of this study was the identification of a molecular signature including various ncRNA biotypes, such as piRNAs, lncRNAs and miRNAs, which may more closely reflect the general biology of underlying renal damage in SLE than use of single markers. Another highlight is the detection of ncRNA targets to construct an interaction regulatory network and identify the gene nodes with an important role in network organization. These findings provide insights into molecular mechanisms and potential targets for treating SLE-associated nephritis. As outlined in our study, a limitation of this study is that the data are sustained by literature evidence in association with renal damage in SLE. Hence, validation methods should be expanded for further verification of the ncRNA signature and potential targets identified, using in vitro studies and techniques such as Western blot or ELISA, to set a benchmark for research into renal damage development in SLE.

In summary, our study identified a plasma exosomal ncRNA profile associated with LN, which targets critical pathways implicated in inflammation, fibrosis, epithelial–mesenchymal transition and actin cytoskeleton, and identifies a handful of potential gene candidates as promising therapeutic targets in LN.

## 4. Materials and Methods

### 4.1. Subjects and Samples

This case-control study included SLE patients and healthy volunteers from the Lupus collection provided by the INCLIVA Biobank (PT20/00029; B.000768 ISCIII), integrated in the Valencian Biobanking and Spanish National Biobanks Networks. Global ncRNA profiling by Small RNA-sequencing included 96 samples from SLE patients, 23 of which with LN, and 25 healthy volunteers. The SLE patients enrolled in the present study met the American College of Rheumatology criteria for SLE. LN was diagnosed following the KDIGO Clinical Practice Guideline for Glomerulonephritis diagnostic criteria, revised in 2017 [43,44]. Healthy control subjects were age- and sex-matched.

### 4.2. Samples

Exosome isolation from plasma samples was performed using a protocol based on sequential ultracentrifugation. All centrifugations were performed at 4 °C using Optima L 100K ultracentrifuge (Beckman Instruments, Brea, CA, USA) [20]. Next, exosome fractions were characterized by the nanoparticle tracking analysis (NTA) on a NanoSight LM10 (Malvern Instrument Ltd., Malvern, UK) and by transmission electron microscopy for double immunogold labeling (CD63 and CD9) (Supplementary Figure S3).

### 4.3. RNA Extraction, Small RNA Library Preparation, and Next-Generation Sequencing

Total RNA was extracted from plasma samples with the miRNeasy Mini Kit (Qiagen, Hilden, Germany) or total Exosome RNA and Protein Isolation Kit (Invitrogen, Life Technologies, Waltham, MA, USA), for plasma exosome samples. RNA amount and purity were quantified by capillary electrophoresis (Agilent 2100 Bioanalyzer, Agilent Technologies, Santa Clara, CA, USA). To normalize the protocol variability, two spike-ins (ath-miR-159a and cel-miR-39) were added to all samples.

Single patient libraries were prepared using Small RNA-Seq Library Prep Kit (Lexogen GmbH, Vienna, Austria) following a small RNA library preparation protocol optimized to very low input samples. Briefly, after adapter ligation and cDNA amplification, libraries were size-selected using Pippin Prep Automated DNA Size Selection platform (Sage science, Beverly, MA, USA), and were purified and concentrated. The quality of individual libraries prior to quantification by real-time quantitative PCR (RT-qPCR) was analyzed by capillary electrophoresis in the QIAxcel Advanced System (Qiagen, Germany). Finally, single libraries were sequenced on the NextSeq 550 platform (Illumina, San Diego, CA, USA), with 2 × 150-cycle paired-end reading mode.

For small-RNA sequencing (RNA-seq), the featureCounts function available in Bioconductor R package Rsubread [45] was used to extract the normalized read count from the smallRNA-seq data, after mapping the trimmed reads by Trim Galore!, using STAR against the last version of the human reference genome (GRCh38) [46]. The mature transcripts of small-RNA species (piRNAs, miRNAs, snRNA, snoRNA and rRNA) were obtained from piRNAbank, miRBase and Ensembl using the featureCounts function. The differential expression levels of small RNAs were calculated with the Bioconductor DESeq2 package for the R software [47]. The raw RNA-Seq dataset can be found at the BioProject repository, (PRJNA904396).

### 4.4. Statistical Analysis

Contrasts between biofluids, plasma and plasma exosomes and between SLE groups were determined by performing a negative binomial generalized log-linear model to analyze the read counts for each gene, adjusted for age and sex. Raw *p*-values were adjusted using the Benjamini–Hochberg procedure, a log2 fold-change of +/− 1.5 was used as an appropriate threshold, and a false discovery rate (FDR) cut-off value of 0.05 was defined as statistically significant, generating enough RNA candidates.

### 4.5. Non-Coding RNA Target Predictions and Functional Enrichment Analysis

The targets for lncRNAs were predicted using LncRRIsearch [48]. The top ten targets for each isoform with an energy threshold >−100 kcal/mol were selected. Three web-based tools were used for miRNA targets: TargetScan, miRDB and miRTarBase. The selection criterion by target was for miRTarBase with a sum of validation methods greater than 1 or if the number of papers was greater than 1. For TargetScan, we set the cumulative weighted context score of <−0.5 and with a Target Score of 90 or higher for miRDB. Predictions overlapping in at least two programs were considered effective targets.

Gene set Over-Representation Analysis (ORA) was performed through GO terms and KEGG pathways in WebGestalt [Gene SeT AnaLysis Toolkit (http://www.webgestalt.org/), access on 15 January 2023], predicting the biological functions and pathways of the candidate target genes involved in ncRNA networks [49]. Each GO and KEGG pathway term with an FDR < 0.05 was defined as significant; the top 20 terms and pathways were selected. The ncRNA targets’ interaction network was generated using the STRING database v11.5. The STRING database provides a confidence score (from 0 to 1) that estimates the likelihood that an annotated interaction between a pair of proteins is biologically meaningful, specific and reproducible. All biological interactions with a confidence score of 0.4 or higher were included. The ncRNA networks were drawn by Cytoscape v3.9.1 using the yFiles organic layout [50].

## Figures and Tables

**Table 1:** Clinical characteristics of study patients’ group.

Variables	Non LN (*n* = 73)	LN (*n* = 23)	CNT (*n* = 25)
Age, years	51.9 ± 11.8	48.7 ± 9.1	40.2 ± 12.0
Female sex. (%)	82.6	87.2	60.7
Serum creatinine (mg/dL)	1.6 ± 7.3	0.8 ± 0.3	0.71 ± 0.11
dsDNA (U/mL)	69.0 ± 92.6	200.7 ± 289.2 **	-
ANA	8.6 ± 4.7	8.2 ± 5.3	-
C3 (mg/dL)	100.1 ± 26.3	87.6 ± 29.6	-
C4 (mg/dL)	20.8 ± 12.4	16.2 ± 10.5	-
SLEDAI index	5.3 ± 5.3	14.3 ± 6.6 **	-
eGFR (CKD-EPI)	98.2 ± 14.4	84.5 ± 30.1 **	105.9 ± 12.5
Log urinary albumin/urinary creatinine	0.62 (0.39–0.89)	2.12 (1.88–2.48) **	0.58 (0.44–0.72)
Basal Treatment (%)			
Hydroxychloroquine	68.5	56.5	
Azathioprine	19.2	17.4	
Prednisone	38.4	47.8	
Methotrexate	6.8	4.3	
Deflazacort	12.3	9.3	
Immunosuppressive therapy	3.7	9.0	
Monoclonal antibodies	4.1	13.0 *	

ANA: antinuclear antibodies; CNT: control; dsDNA: double strand DNA; eGFR: estimated glomerular filtration rate; LN: lupus nephropathy; SLEDAI: systemic lupus erythematosus disease activity index. * *p* < 0.05; ** *p* < 0.01. Continuous variables are presented as mean ± standard deviation. Urinary albumin excretion and urinary albumin/urinary creatinine are presented as median and interquartile range and categorical variables as percentage (%).

## Data Availability

The raw RNA-Seq dataset is available at the BioProject repository, accession: PRJNA904396.

## Supplementary Materials

The following supporting information can be downloaded at: https://www.mdpi.com/article/10.3390/ijms24087088/s1.
