# Supplementary material for: Plasma Exosomal Non-Coding RNA Profile Associated with Renal Damage Reveals Potential Therapeutic Targets in Lupus Nephritis
Source: Int J Mol Sci. 2023 Apr 11;24(8):7088. doi: 10.3390/ijms24087088 (PMC10139178; doi:10.3390/ijms24087088)
Supplement: Supplementary file 1 [file ijms-24-07088-s001.zip › ijms-2301342-supplementary.pdf]

Supplemental material

Plasma exosomal non-coding RNA profile associated to renal damage reveals potential therapeutic targets in lupus nephritis

Ana Flores-Chova [1,†], Olga Martinez-Arroyo [1,†], Angela L Riffo-Campos [2,3], Ana Ortega [1,4], Maria Jose Forner[5,6,‡] and Raquel Cortes [2,‡,*]

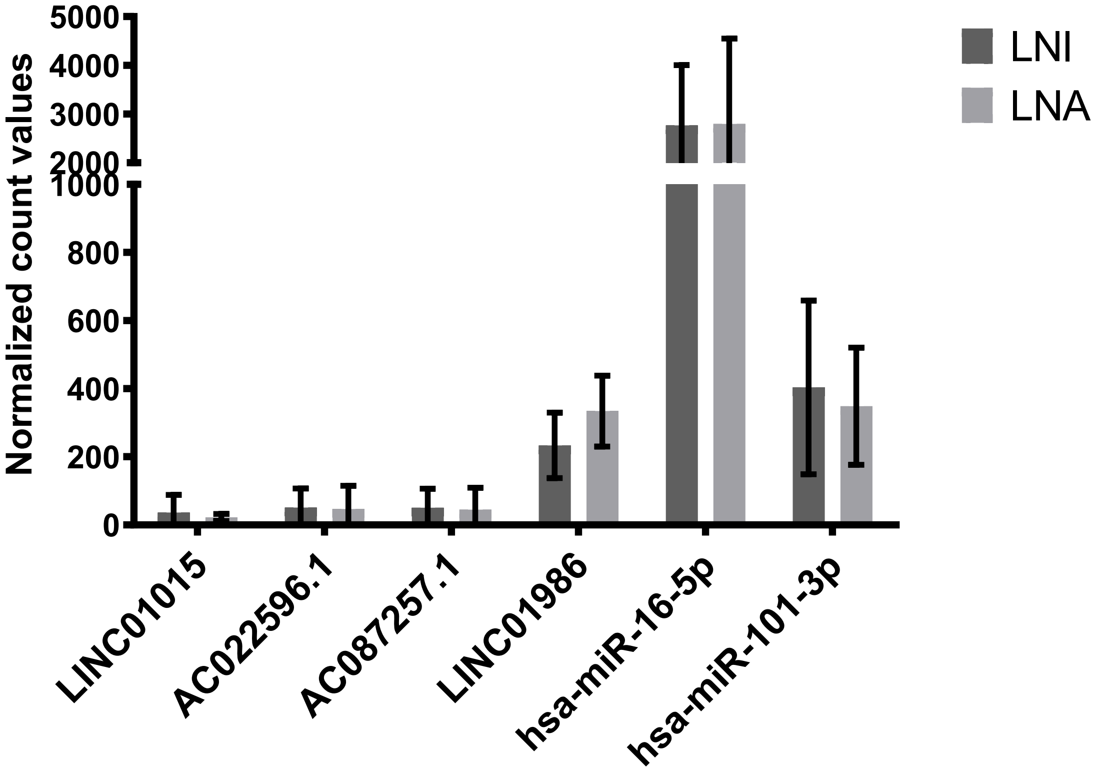

Figure S1. Normalized count values of ncRNA according to disease activity in lupus nephritis group. Comparison of ncRNA read counts in lupus nephritis (LN) according to low disease activity (LNI) or high (LNA). No statistical significant differences were found to compare by U-Mann-Whitney non-parametric test.

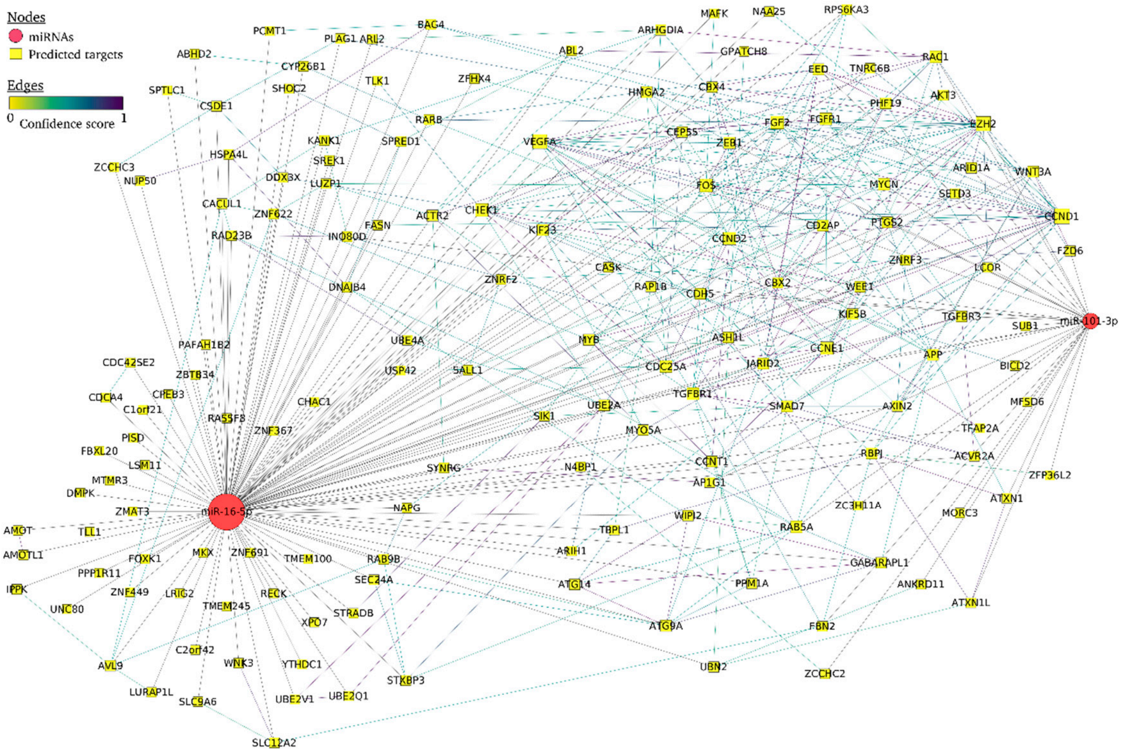

Figure S2. Overview of the plasma exosomes microRNA–mRNA target interaction network related to lupus nephritis. Each miRNA is a red circle node; miRNA targets are yellow square nodes. The node size increases in relation to the number of edges (network degree), and a higher confidence score indicates a darker edge between nodes.

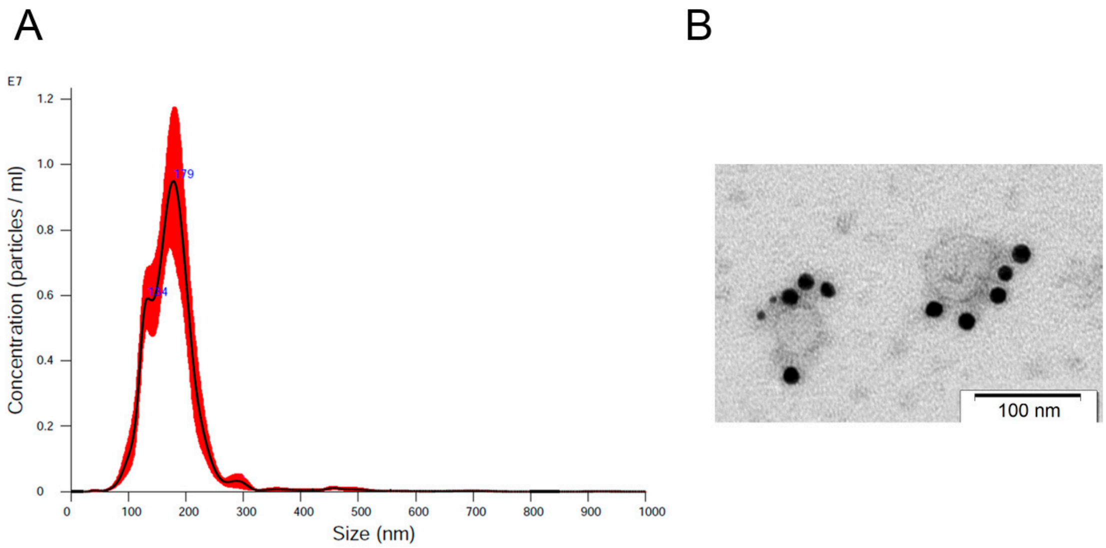

Figure S3. Characterization of plasma exosome enriched-fraction. A) Graph showing the NTA results with concentration and size of particles. B) Transmission electron microscopy micrographs of CD9 (15 nm) and CD63 (6 nm) with immunogold labeling.

Supplemental Table S1. List of differentially expressed ncRNA in plasma exosomes from patients with (n=29 non-coding RNA) or without lupus nephritis (n=36 non-coding RNA)

**LN group**					**Non LN group**				
**Up-regulated**					**Up-regulated**				
**ID**	**SYMBOL**	**BIOTYPE**	**Log2 FC**	**FDR**	**ID**	**SYMBOL**	**BIOTYPE**	**Log2 FC**	**FDR**
ENSG00000229852	AC019205.1	lncRNA	5,093	0,007	ENSG00000229852	AC019205.1	lncRNA	4,19	0,0003
ENSG00000224582	LINC01015	lncRNA	5,075	0,022		hsa-miR-652-3p	miRNA	3,5	0,0002
	hsa-piR-020244	piRNA	4,141	0,015		hsa-miR-19a-3p	miRNA	2,78	0,0062
ENSG00000288088	AL606923.2	lncRNA	3,625	0,027	ENSG00000288088	AL606923.2	lncRNA	2,66	0,0384
ENSG00000234570	ZFRP1	pseudogene	3,617	0,035	ENSG00000286269	AC092661.1	lncRNA	2,6	0,0009
ENSG00000286269	AC092661.1	lncRNA	2,501	0,052	ENSG00000237737	DCTN1-AS1	lncRNA	2,37	0,0050
	hsa-miR-107	miRNA	1,860	0,000		hsa-miR-106b-5p	miRNA	2,35	0,0077
	hsa-miR-451a	miRNA	1,743	0,000	ENSG00000229140	CCDC26	lncRNA	2,07	0,0288
	hsa-miR-15a-5p	miRNA	1,679	0,006		hsa-miR-20a-5p	miRNA	2,06	0,0375
	hsa-miR-144-5p	miRNA	1,624	0,006		hsa-miR-144-3p	miRNA	1,89	0,0000
ENSG00000264729	AC022596.1	lncRNA	1,410	0,025	ENSG00000227733	AC239809.3	lncRNA	1,73	0,0178
ENSG00000189423	USP32P3	pseudogene	1,404	0,025	ENSG00000205746	PKD1P4	pseudogene	1,71	0,0145
ENSG00000248100	AC087257.1	lncRNA	1,329	0,033		hsa-miR-107	miRNA	1,7	0,0002
	hsa-piR-013323	piRNA	1,236	0,025		hsa-miR-629-5p	miRNA	1,69	0,0371
	hsa-miR-16-5p	miRNA	1,235	0,006		hsa-miR-144-5p	miRNA	1,63	0,0027
	hsa-miR-144-3p	miRNA	1,189	0,033		hsa-miR-451a	miRNA	1,62	0,0001
ENSG00000223587	LINC01986	lncRNA	1,079	0,010		hsa-miR-15a-5p	miRNA	1,58	0,0008
	hsa-miR-10b-5p	miRNA	1,020	0,013	ENSG00000254681	PKD1P5	pseudogene	1,58	0,0144
	hsa-miR-25-3p	miRNA	1,000	0,044	ENSG00000227827	PKD1P2	pseudogene	1,45	0,0249
	hsa-miR-101-3p	miRNA	0,949	0,054		hsa-miR-25-3p	miRNA	1,4	0,0023
	hsa-piR-009000	piRNA	0,926	0,033		hsa-miR-185-5p	miRNA	1,34	0,0027

	hsa-piR-020364	piRNA	0,918	0,025		hsa-miR-130a-3p	miRNA	1,29	0,0380
ENSG00000274602	PI4KAP1	pseudogene	0,819	0,044	ENSG00000201098	RNY1	Y-RNA	1,27	0,0135
ENSG00000183506	PI4KAP2	pseudogene	0,819	0,044		hsa-miR-143-3p	miRNA	1,22	0,0060
**Down-regulated**						hsa-miR-16-2-3p	miRNA	1,2	0,0248
	hsa-piR-001184	piRNA	-0,995	0,016		hsa-miR-10b-5p	miRNA	1,06	0,0010
ENSG00000172297	GOLGA2P3Y	pseudogene	-1,180	0,033	ENSG00000252487	RNY4P20	Y-RNA	1,06	0,0278
ENSG00000259570	AC243562.1	pseudogene	-1,180	0,033		hsa-miR-30a-5p	miRNA	1,04	0,0133
ENSG00000239533	GOLGA2P2Y	pseudogene	-1,180	0,033		hsa-miR-10a-5p	miRNA	0,82	0,0192
ENSG00000225151	GOLGA2P7	pseudogene	-1,180	0,033	**Down-regulated**				
						hsa_piR_018624	piRNA	-0,46	0,0307
						hsa_piR_019949	piRNA	-1	0,0011
					ENSG00000259570	AC243562.1	pseudogene	-1,45	0,0027
					ENSG00000239533	GOLGA2P2Y	pseudogene	-1,45	0,0027
					ENSG00000172297	GOLGA2P3Y	pseudogene	-1,45	0,0027
					ENSG00000225151	GOLGA2P7	pseudogene	-1,45	0,0028
					ENSG00000234564	HSPA8P20	pseudogene	-2,97	0,0202

Table S2. List of the microRNA and lncRNA targets in plasma exosomes from LN patients.

Exo-P miRNA targets		Exo-P lncNA targets	
ABHD2	Protein Coding	ABI2	Protein Coding
ABL2	Protein Coding	AHCTF1	Protein Coding
ACTR2	Protein Coding	AP006621.9	lncRNA
ACVR2A	Protein Coding	ARHGEF35	Protein Coding
AKT3	Protein Coding	ARHGEF5	Protein Coding
AMOT	Protein Coding	B3GALNT2	Protein Coding
AMOTL1	Protein Coding	CCDC85C	Protein Coding
ANKRD11	Protein Coding	CENPB	Protein Coding
AP1G1	Protein Coding	CHP2	Protein Coding
APP	Protein Coding	EMC10	Protein Coding
ARHGDIA	Protein Coding	FAM111A	Protein Coding
ARID1A	Protein Coding	FAM98B	Protein Coding
ARIH1	Protein Coding	FPGT-TNNI3K	Protein Coding
ARL2	Protein Coding	GMDS-AS1	lncRNA
ASH1L	Protein Coding	GOLGA6L22	Protein Coding
ATG14	Protein Coding	GOLGA6L6	Protein Coding
ATG9A	Protein Coding	GPR68	Protein Coding
ATXN1	Protein Coding	HHIPL1	Protein Coding
ATXN1L	Protein Coding	IGF2	Protein Coding
AVL9	Protein Coding	KIAA1429	Protein Coding
AXIN2	Protein Coding	NR2F1-AS1	lncRNA
BAG4	Protein Coding	OSBPL6	Protein Coding
BICD2	Protein Coding	PARVG	Protein Coding
C1orf21	Protein Coding	RP11-133K1.7	lncRNA
C2orf42	Protein Coding	RP11-384G23.1	lncRNA

CACUL1	Protein Coding	RP11-573D15.8	lncRNA
CASK	Protein Coding	RP11-95O2.5	lncRNA
CBX2	Protein Coding	RP1-278O22.2	lncRNA
CBX4	Protein Coding	RP3-323A16.1	lncRNA
CCND1	Protein Coding	RP4-671O14.6	lncRNA
CCND2	Protein Coding	RPGR	Protein Coding
CCNE1	Protein Coding	SNX8	Protein Coding
CCNT1	Protein Coding	TNNI3K	Protein Coding
CD2AP	Protein Coding	UQCC1	Protein Coding
CDC25A	Protein Coding		
CDC42SE2	Protein Coding		
CDCA4	Protein Coding		
CDH5	Protein Coding		
CEP55	Protein Coding		
CHAC1	Protein Coding		
CHEK1	Protein Coding		
CPEB3	Protein Coding		
CSDE1	Protein Coding		
CYP26B1	Protein Coding		
DDX3X	Protein Coding		
DMPK	Protein Coding		
DNAJB4	Protein Coding		
EED	Protein Coding		
EZH2	Protein Coding		
FASN	Protein Coding		
FBN2	Protein Coding		
FBXL20	Protein Coding		

FGF2	Protein Coding		
FGFR1	Protein Coding		
FOS	Protein Coding		
FOXK1	Protein Coding		
FZD6	Protein Coding		
GABARAPL1	Protein Coding		
GPATCH8	Protein Coding		
HMGA2	Protein Coding		
HSPA4L	Protein Coding		
INO80D	Protein Coding		
IPPK	Protein Coding		
JARID2	Protein Coding		
KANK1	Protein Coding		
KIF23	Protein Coding		
KIF5B	Protein Coding		
LCOR	Protein Coding		
LRIG2	Protein Coding		
LSM11	Protein Coding		
LURAP1L	Protein Coding		
LUZP1	Protein Coding		
MAFK	Protein Coding		
MFSD6	Protein Coding		
MKX	Protein Coding		
MORC3	Protein Coding		
MTMR3	Protein Coding		
MYB	Protein Coding		
MYCN	Protein Coding		

MYO5A	Protein Coding		
N4BP1	Protein Coding		
NAA25	Protein Coding		
NAPG	Protein Coding		
NUP50	Protein Coding		
PAFAH1B2	Protein Coding		
PCMT1	Protein Coding		
PHF19	Protein Coding		
PISD	Protein Coding		
PLAG1	Protein Coding		
PPM1A	Protein Coding		
PPP1R11	Protein Coding		
PTGS2	Protein Coding		
RAB5A	Protein Coding		
RAB9B	Protein Coding		
RAC1	Protein Coding		
RAD23B	Protein Coding		
RAP1B	Protein Coding		
RARB	Protein Coding		
RASSF8	Protein Coding		
RBPJ	Protein Coding		
RECK	Protein Coding		
RPS6KA3	Protein Coding		
SALL1	Protein Coding		
SEC24A	Protein Coding		
SETD3	Protein Coding		
SHOC2	Protein Coding		

SIK1	Protein Coding		
SLC12A2	Protein Coding		
SLC9A6	Protein Coding		
SMAD7	Protein Coding		
SPRED1	Protein Coding		
SPTLC1	Protein Coding		
SREK1	Protein Coding		
STRADB	Protein Coding		
STXBP3	Protein Coding		
SUB1	Protein Coding		
SYNRG	Protein Coding		
TBPL1	Protein Coding		
TFAP2A	Protein Coding		
TGFBR1	Protein Coding		
TGFBR3	Protein Coding		
TLK1	Protein Coding		
TLL1	Protein Coding		
TMEM100	Protein Coding		
TMEM245	Protein Coding		
TNRC6B	Protein Coding		
UBE2A	Protein Coding		
UBE2Q1	Protein Coding		
UBE2V1	Protein Coding		
UBE4A	Protein Coding		
UBN2	Protein Coding		
UNC80	Protein Coding		
USP42	Protein Coding		

VEGFA	Protein Coding		
WEE1	Protein Coding		
WIPI2	Protein Coding		
WNK3	Protein Coding		
WNT3A	Protein Coding		
XPO7	Protein Coding		
YTHDC1	Protein Coding		
ZBTB34	Protein Coding		
ZC3H11A	Protein Coding		
ZCCHC2	Protein Coding		
ZCCHC3	Protein Coding		
ZEB1	Protein Coding		
ZFHX4	Protein Coding		
ZFP36L2	Protein Coding		
ZMAT3	Protein Coding		
ZNF367	Protein Coding		
ZNF449	Protein Coding		
ZNF622	Protein Coding		
ZNF691	Protein Coding		
ZNRF2	Protein Coding		
ZNRF3	Protein Coding		
